# Finding the right balance: implementation of public–private partnership in artemisinin-based combination therapy provision in Manokwari, Indonesia

**DOI:** 10.1186/s40545-021-00347-2

**Published:** 2021-11-16

**Authors:** Astri Ferdiana, Utsamani Cintyamena, Luthfi Azizatunnisa’, Edi Sunandar, Ari Probandari

**Affiliations:** 1 Center for Tropical Medicine, Faculty of Medicine, Public Health and Nursing, Universitas Gadjah Mada, Yogyakarta, Indonesia; 2 Department of Public Health, Faculty of Medicine, Universitas Mataram, Mataram, Indonesia; 3 Department of Health Behavior, Environment and Social Medicine, Faculty of Medicine, Public Health and Nursing, Universitas Gadjah Mada, Yogyakarta, Indonesia; 4 Provincial Health Office of West Papua, Manokwari, Indonesia; 5 Department of Public Health, Faculty of Medicine, Universitas Sebelas Maret, Surakarta, Indonesia

**Keywords:** Indonesia, Malaria, Private sector, Pharmacies, Public–private partnership

## Abstract

**Background:**

Indonesia is the second country with the highest number of malaria cases in Southeast Asia. Private health providers including community pharmacies often become the first point of care for the population seeking malaria treatment; however, public–private partnerships for malaria control are not widely implemented. This paper explores the acceptability of  a public-private partnership program on the  provision of subsidized artemisinin-based combination therapies (ACTs) in community pharmacies from the perspectives of private health providers, patients, and program implementers.

**Methods:**

The study was conducted in Manokwari District in West Papua Province, one of the highest endemic districts in Indonesia. Qualitative methods using interviews and focus group discussions (FGDs) were employed to explore the following dimensions of acceptability: affective attitude, burden, ethicality, intervention coherence, opportunity cost, perceived effectiveness, and self-efficacy. Study participants were program implementers, private health providers, and pharmacy clients. Audio-recorded interviews were transcribed and analyzed using thematic analysis. Secondary data on malaria cases and the use of ACTs reported by community pharmacies were also recorded.

**Results:**

Only one-fourth of the total community pharmacies in Manokwari participated in the partnership, suggesting low coverage of the program. The proportion of malaria cases reported by community pharmacies increased from 6.9% in 2018 to 30.7% of cases. Most participants had a positive attitude towards the program, which might be associated with the perceived effectiveness of the partnership in improving access to ACTs. Despite the good understanding of the intervention by the participating pharmacies, limited involvement of private physicians often resulted in non-standardized treatment practices. The partnership also imposed a burden on private health providers in terms of human resources and time which entailed significant opportunity costs. A number of ethical issues might undermine the equity of access to ACTs.

**Conclusion:**

Despite the positive attitude to the partnership, the perceived burden might outweigh the tangible benefits, posing threats to scaling up the intervention and sustainability. Innovations to simplify the administrative procedures in combination with performance-based incentives are needed to improve implementation. Engagement of patients and physicians is needed to increase the effectiveness of the partnership.

## Introduction

Since 2010, artemisinin-based combination therapies (ACTs) have been recommended by the WHO as a standard treatment for uncomplicated malaria [1]. After parasitological confirmation of diagnosis, ACTs are initiated either through rapid diagnostic tests or microscopy [1]. ACTs are typically highly subsidized or provided at no cost to patients in the public sector when the diagnosis and treatment have been established according to standard guidelines [2]. In private drug outlets such as community pharmacies, however, similar care standards are often not available.

In many malaria-endemic countries, community pharmacies are often the first and only source of care [3]. Seeking treatment for fever or malaria symptoms at community pharmacies is particularly common among urban and rural populations because these providers are more accessible, especially in developing and low-income countries in Asia, Africa, and South America [4, 5]. Nevertheless, antimalarial drugs in community pharmacies are often dispensed without laboratory confirmation of diagnosis [6]. Non-standard treatments such as quinine are still dispensed because ACTs are not available nor affordable. Consequently, the use of ACTs as the first line of treatment for uncomplicated malaria is limited. Patients who seek treatment in the private sector thus may be using ineffective medication [2].

Given the large proportion of patients seeking care at private health providers, implementing a public–private partnership may help in fast-tracking malaria elimination [7]. Universal access to quality malaria diagnosis and treatment must be ensured in both the public and private health sectors. Globally, the collaboration between the government and private sectors in malaria control has started in the late 1990s [8]. The existing public and private sector collaboration in malaria control mostly focus on the provision of antimalarial drugs and diagnostic tests, indoor residual spraying, and insecticide mosquito net [8, 9]. The partnership between public and private on ACTs provision has been widely implemented in several sub-Saharan countries, yet little evidence exists for its implementation in the Southeast Asian region. Although the incidence rate of malaria in this region has dropped by 70% between 2018 and 2010, several countries including Indonesia still faced challenges to achieve elimination [10].

Indonesia is the second country with the largest number of malaria cases within the region (30%) and contributes 5% of malaria vivax cases globally [10]. In 2018, a total of 180,205 positive cases of malaria were reported. The annual parasite incidence (API) in 2018 was 0.68/1000 population [11]. Although more than half of 514 districts in Indonesia have been declared malaria-free, malaria remains highly endemic in some areas, including the West Papua Province [12]. Within this province, the highest number of malaria cases is found in Manokwari District, the province’s capital. In 2018, 4340 malaria cases were found with API of 24.1 per 1,000 population. This district aims to achieve malaria elimination by 2025.

Partnership with the private sector has been emphasized in the Indonesian national strategy for malaria elimination, including establishing collaboration with community pharmacies to provide access to antimalarial drugs [12]. A previous study in Papua, the highest malaria-endemic area in Indonesia, reported that 51% of patients with malaria obtained antimalarial from private health providers [13], suggesting that private health providers are an important source of care for malaria. However, partnerships with the private sector in malaria control have only been implemented in a few endemic districts in Indonesia, including Manokwari.

**Fig. 1:**
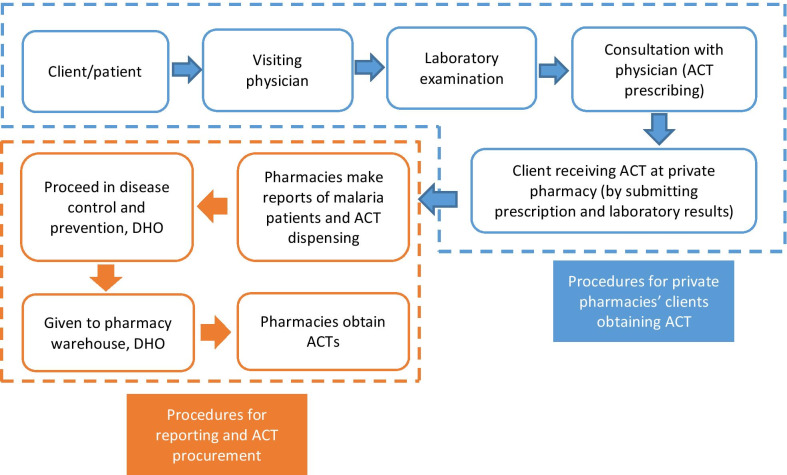
Program flowchart

The public–private partnership in malaria control in Manokwari was established in 2012 and aimed at involving private health providers in the diagnosis and treatment of malaria based on the national guidelines. Private health providers were invited to participate in this partnership on a voluntary basis. In 2018, a memorandum of understanding (MoU) on public–private partnership in malaria was created between the Manokwari District Health Office (DHO) and community pharmacies. In this partnership, ACTs are provided in the pharmacies at no cost by DHO, provided that community pharmacies report data on the number of cases and ACTs dispensed. Community pharmacies are allowed to charge a maximum administrative fee of IDR 10,000 (equivalent to USD 0.75). The DHO provides ACTs for free through the existing logistics and supply channels of the public sector. The community pharmacies can only dispense ACTs to patients upon physician’s prescription and laboratory confirmation. In order to restock, pharmacists fill in a form for submission to the DHO. The physician prescription and laboratory confirmation result must be attached to this request form. The DHO would then send a request letter to the district pharmacy warehouse so that the pharmacists can collect the requested ACTs. Figure 1 explains the mechanism of the ACTs provision in the pharmacies through DHO channel.

Despite having been implemented for some time, it is unknown whether the partnership program is well accepted by private health providers especially community pharmacies and physicians, program implementers and patients. Assessing the acceptability of the program will contribute to the improvement of the program implementation, as well as program sustainability and the possibility of scaling up [14]. To date, empirical evidence on the implementation of public–private partnership in malaria in Indonesia is scarce.

This paper explores the acceptability of public–private partnership for ACTs provision as perceived by the private health providers, patients, and program implementers in Manokwari District, West Papua province, Indonesia.

## Methods

### Setting

Manokwari district is a semi-urban area inhabited by approximately 200,000 people. Healthcare services include 13 community health centers (*Puskesmas*), one district public hospital, and three military hospitals. The number of community pharmacies in Manokwari had grown from 68 in 2017, 78 in 2018 to 86 in 2019 [15, 16]. Most have a clinical laboratory located within the compound.

In Indonesia, there are around 28,000 pharmacies [17]. These business entities are allowed to sell prescription and over-the-counter medicine and must be attended by a registered pharmacist. The professional license of pharmacists and operational license of the pharmacies are issued by the DHO upon the local professional associations’ recommendation [18].

### Study design

The study employed qualitative methods to develop a complete understanding of stakeholder perceptions around program acceptability.

**Fig. 2:**
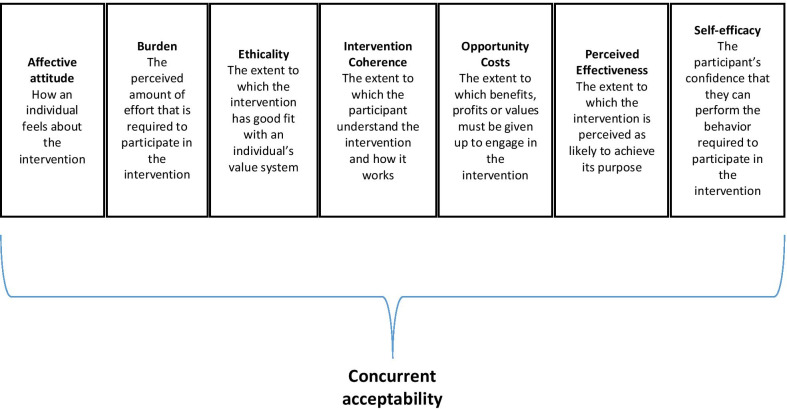
Framework of acceptability

We define acceptability as “a multi-faceted construct that reflects the extent to which people delivering or receiving a healthcare intervention consider it to be appropriate, based on anticipated or experienced cognitive and emotional responses to the intervention” [14]. In understanding how the acceptability of the intervention works, we used the theoretical framework of acceptability (TFA) (Fig. 2) [14]. The TFA is a comprehensive framework that proposes that acceptability’s core dimensions include behavior, affection and cognition [14, 19]. This framework has been used in complex program evaluations to assess ‘acceptability’, such as mental health promotion programs, physical activity in postnatal group programs, and chronic kidney patients [20–22].

A combination of focus group discussions (FGDs) and interviews were used to explore the following dimensions of acceptability according to the TFA framework: affective attitude, burden, ethicality, intervention coherence, opportunity cost, perceived effectiveness, and self-efficacy [14]. The dimensions of acceptability are shown in Table 1.

**Table 1:** Dimensions of acceptability and the definition based on Sekhon et al. (2017)

Dimension	Definition
Affective attitude	How an individual feels about the intervention
Burden	The perceived amount of effort that is required to participate in the intervention
Ethicality	The extent to which the intervention has good fit with an individual’s value system
Intervention coherence	The extent to which the participant understands the intervention and how it works
Opportunity cost	The extent to which benefits, profits or values must be given up to engage in the intervention
Perceived effectiveness	The extent to which the intervention is perceived as likely to achieve its purpose
Self-efficacy	The participant’s confidence that they can perform the behavior required to participate in the intervention

### Participants and recruitment

Purposive sampling was employed to select the following groups of participants: (1) program implementers and representatives of professional associations; (2) private health providers and (3) community pharmacy clients.

Relevant program implementers were selected from the disease control as well as pharmaceutical and health services sections, since the management of antimalarial drugs involved both. Representatives of professional associations were also included. Eligible participants were contacted via phone calls using contact information obtained from the provincial health office.

Private health providers included community pharmacy staff, physicians, and laboratory analysts that had been working for more than 6 months. Community pharmacy staff must have a responsibility for antimalarial drug dispensing or management, while physicians must have treated a malaria patient in his/her practice. Eligible participants were identified from the list of private health providers at DHO and contacted by an invitation letter containing the study information sheet and consent form. The invitations were followed up by phone or text messages within 2–3 days to confirm participation and schedule interview appointments.

Clients at community pharmacies were selected from those aged ≥ 18 years, obtained antimalarials for themselves or for family members and resided in the study area for more than 6 months. At first, we included only malaria-positive clients aged ≥ 18 years, obtaining antimalarials at community pharmacies. However, we expanded the inclusion criteria to include patients aged < 18 years. We also revised the inclusion criteria from being confirmed malaria positive to those who obtained antimalarials at community pharmacies to capture those who purchased antimalarials without laboratory confirmation. These changes have been approved by the ethics committee.

To recruit clients, research assistants visited community pharmacies participating in the partnership and asked the attending staff to inform any client who came to obtain antimalarials about the study. Eligible participants who agreed to participate were asked to provide their contact information, which was then passed on to the research team. The research assistants then contacted the clients and made interview appointments.

### Data collection

The following topics were explored during interviews: awareness and initiation of the partnership, perceived benefits and disadvantages, factors that prevented or facilitated implementation, and perceived impacts. In the FGDs, the following topics were explored using a topic list: awareness and perception of the partnership, implementation process, as well as factors affecting the implementation of the partnership. Probing questions were asked to understand further how the participants experienced and implemented the partnership.

All interviews with program implementers and private health providers took place in a private location, mostly at the participant’s workplace. Interviews lasted between 45 and 60 min. Interviews with pharmacy clients were conducted at the community pharmacies or at home, depending on participant preference. Interviews took around 20–30 min for each participant.

Two local research assistants were recruited and trained in qualitative data collection methods. FGDs were conducted by AF, AP and ES. The few first interviews were conducted by a team of two, i.e., one researcher and one research assistant, to ensure a similar understanding and sufficient probing. Local research assistants conducted the rest of the interviews. All interviews were conducted in Indonesian language or the local dialect.

### Data analysis

All interviews and FGDs were audio-recorded and transcribed verbatim by a professional transcriber. Transcripts were read by all researchers and analyzed using thematic analysis based on the pre-determined constructs of acceptability. All researchers read the transcripts line by line and developed codes. One researcher (LA) compiled and compared the codes. Any discrepancy was discussed among the researchers. Recurrent codes were summarized into subthemes and subsequently themes. Themes and representative quotes were identified and recorded using Excel sheets. Representative quotes were translated into the English language.

### Ethics

Ethical approval was sought from the Medical and Health Research Ethical Committee of the Faculty of Medicine Universitas Gadjah Mada (KE/FK/0917/EC/2019). Ethical approval was also obtained from the World Health Organization’s Ethics Review Committee. One amendment of the inclusion criteria was requested and approved accordingly.

Prior to data collection, the study objectives and procedures were explained to the participants. Private health providers were told that their participation in the study and responses would not affect their relationship with the district health office in any way. All participants were asked to sign written informed consent in the Indonesian language before their participation in the study. For clients aged < 18 years, consent was first obtained from the patients’ parents or guardians before interviews.

## Results

### Characteristics of participants

A total of 11 interviews were conducted with policymakers or program implementers, consisting of 8 program implementers at the district level and 3 representatives from professional associations. The interview with the program implementers at the food and drug administration was conducted in a group due to participant preference.

Twenty-eight private health providers from 21 community pharmacies were interviewed, consisting of 7 physicians, 15 pharmacists, 4 owners of pharmacies, and 2 laboratory analysts. Twenty-one participants were female. Age ranged from 23 to 60 years. Eighteen pharmacies participated in the partnership, while 3 did not. Table 2 summarizes the characteristics of interview participants.

**Table 2:** Characteristics of key informant interview respondents

Characteristics	*N*
Type of participants (*n* = 11)	
Program implementer at provincial health office	0
Program implementer at district health office	4
Program implementer at food and drug administration	4
Representatives of professional association	3
Gender	
Female	5
Male	6
Age range (min–max)	29–56

A total of 9 client participants from 7 community pharmacies were interviewed. Seven were female, and 2 were male. Eight participants were the patient themselves, and one was the caregiver.

Three FGDs were conducted with 23 participants from community pharmacies participating in the partnership. The majority of participants were pharmacists and female. Their characteristics are presented in Table 3.

**Table 3:** Characteristics of focus group discussion respondents

Characteristics	*N*
Type of staff (*n* = 23)	
Pharmacist	18
Pharmacy assistant	2
Staff	2
Manager	1
Gender (*n* = 23)	
Female	18
Male	5
Type of pharmacies (*n* = 23)	
MoU	23
Non-MoU	0

### Program implementation

Since its inception, 34 community pharmacies have participated in the partnership program. Out of these, only 20 pharmacies regularly sourced antimalarials from the DHO. Data showed that in 2018, a total of 342 out of 4985 cases (6.9%) were reported from private health providers, while in 2019, 1452 out of 5176 (30.7%) cases were reported.

### Acceptability of program

The results of the interview were organized according to the dimensions of acceptability. These dimensions were organized as follows: perceived effectiveness, affective attitude, intervention coherence, self-efficacy, perceived burden, opportunity costs, and ethicality [14].

#### Perceived effectiveness

Most participants perceived that the partnership involving community pharmacies increase community access to antimalarial drugs. The provision of ACTs at community pharmacies at no cost would reduce financial barriers of access.

Well, if the government involved the private sector, it is even better. It means drug availability will be better monitored. Moreover, people will be less burdened with a relatively high medical cost. Patent drugs are quite expensive (DHO, interview)

It was perceived that adequate ACT supply to community pharmacies was guaranteed by pharmacies’ participation in the partnership and better monitored, thereby ensuring community access to ACTs.

Well, with this partnership, health facilities can have consistent drugs supply. If our stock is nearly out, we can make a request to DHO. So, it helps us to give better services to the community (Pharmacist 1, interview)

Participants also agreed with the partnership's aim and content to ensure the quality of care of malaria. Within the partnership, ACTs can only be administered with diagnostic confirmation and physician's prescription. Participants mentioned that because malaria is highly endemic in Manokwari, it was very common for patients to directly come to the laboratory, ask for a blood test, and bring the result to pharmacies to dispense antimalarials. As ACTs were not always available at pharmacies, antimalarials given were not in line with the national guideline which asserted that ACTs be given to uncomplicated malaria as the first line of treatment.

I am very happy that the government procures malaria drugs (to community pharmacies) because the demand is actually very high. Sometimes patients who come to the pharmacy bring lab test result, but they do not want to consult to physician. Maybe it will cost them more (if they consult a physician). They just want to consult to the pharmacist, and they usually get primaquine (Physician 1, interview)

The partnership was also expected to improve private physician adherence to the malaria guidelines when prescribing antimalarials.

If there is no partnership, the private provider will be running services without standard. This MoU engaged them to improve adherence of physician to implement malaria treatment guidelines (Professional association 1, interview)

Program implementers reported that the partnership allowed data collection from the private physician practices which were formerly difficult to obtain, thus allow better monitoring of district malaria prevalence. It was agreed among the program implementers that the partnership could potentially contribute to malaria elimination and greatly reduce the burden of the public sector in malaria control.

#### Affective attitude

Most pharmacist participants expressed positive attitude towards the partnership. The perceived effectiveness of the program and social motivation to contribute to malaria elimination has driven community pharmacies to invest time in the partnership. They were well aware of the significance of malaria as a long-standing public health problem in Manokwari. The feeling of moral responsibility to contribute to malaria elimination created motivation to participate in the partnership that by joining the partnership, they would personally be able to support the government in antimalarial drug distribution to the community.

…for us [pharmacists], actually we want to help people, so we facilitate them to get free medicine, we want to help. (Pharmacist, FGD 3)

Physician participants also expressed positive attitude and support towards the partnership as it would ensure that ACTs are available and affordable, especially for the poor community members.

Client participants also had a positive perception towards the provision of antimalarials at the community pharmacies, especially as clients often experienced difficulties in obtaining antimalarials at the community pharmacies.

#### Intervention coherence

Most community pharmacy staff described that they would be able to request ACTs at no cost from the district warehouse only when they submitted patient data, including laboratory test results and prescription copies. This suggested a good understanding of the intervention, especially on the procurement component.

However, the management of antimalarial stock varied between pharmacies. Some pharmacies documented the antimalarial stock separately from other antibiotics and managed by one designated staff member, usually the pharmacist.

If we take medicine from there [DHO], in pharmacies, we write again in the drug entry record. In our pharmacy, we separate the storage for antimalarial drugs, so we can easily see the cycle. Thus, when the drugs are available, we also record patients according to logmal [logistic malaria] and regmal [register malaria] (Pharmacist, FGD3)

Pharmacies reported that only patients who presented with malaria positive tests and prescriptions would be given ACTs, suggesting good adherence to the procedure despite the absence of a standard operating procedure (SOP) or implementation guideline. However, the absence of a guideline created different practices of ACT dispensing between pharmacies when the ACTs were out of stock. Pharmacies would dispense different drugs according to their knowledge and experience rather than following the national guideline.

In addition, the practice of prescription analysis prior to dispensing antimalarial drugs was also different between pharmacies. Some pharmacists would assess the appropriateness of dosage when they received the prescription. When the dosage was not in line with the national guideline, they would contact the prescribing physician for clarification. However, such practices were not implemented in other pharmacies.

Despite the pharmacies’ adherence to the intervention, the prescription practices among physicians varied. It was reported by pharmacist participants that a number of patients with positive laboratory confirmation were still prescribed non-ACTs by physicians. Some patients were still given antimalarials based on clinical diagnosis only.

The patient lab result was negative, but the physician prescribed him/her with ACT. So far, I heard that in hospitals, Puskesmas (primary health center), or clinics, physician diagnosis of malaria is still based on the clinical symptoms, for instance, fever and other malaria symptoms, and physician decides to give antimalarial drugs. After the lab examination, turned out she/he was negative. Meanwhile, the procedure to give ACT is not like that; lab result has to be positive. We often experienced misunderstandings with the physician. (Pharmacist, FGD2)

Several physician participants, however, mentioned that they were not informed regarding the partnership between community pharmacies and DHO. In fact, a few participants first heard about the partnership at the time of the interview.

So far, I do not really understand about it. What kind of partnership? I have not received any information. I never heard about that partnership with the private sector (Physician 1, interview)

#### Self-efficacy

Program implementers participants expressed their confidence in implementing this partnership. They perceived that the partnership had been well implemented.

In our opinion, our capacity to implement this partnership is great because, with this partnership, we have worked together with private provider (DHO, interview)

On the contrary, private health provider participants were not confident enough that other pharmacists or pharmacy assistants would follow the procedures.

From my point of view, the SOP will be implemented, but you know in Indonesia, people often difficult to follow the procedures. If the SOP is already developed, we have to slowly implement it and make it as our habit. For this condition, I think SOP is very important (Pharmacist, FGD 3)

#### Perceived burden

Pharmacist participants reported that they often felt burdened by joining the partnership, especially as they lack the human resources and time to manage the reporting and procurement process. The procedure to obtain ACTs was perceived to be cumbersome; the DHO malaria officer must first approve the request, however, this person was sometimes not present at the DHO office. After obtaining the approval, the pharmacy staff had to go to the pharmacy warehouse to collect the medicine. It was not possible to collect a large number of medications at once; hence, the staff had to go to the DHO more than once a month.

The reporting is a burden for us… because in our pharmacy we had to take care many things. Administration problems are many; items come in, invoices are a burden. Moreover, we need to ask the drug there, and we need to give reports, and the report that we gave is not perfect; I mean it was not as what they want, and they asked us to revise it again. Then we need to submit there again. (Pharmacist 6, interview)

Financial incentives for community pharmacies were in fact almost none, as malaria patients that purchased antimalarials were only required to pay for IDR 5000–10,000 for administrative fees. This small amount was used for buying consumables such as drug labels and plastic wraps. This, however, raised some debates among the pharmacists, as it could lead to ethical and legal issues.

The pharmacy management asked to charge for administrative cost. It was not much, only for providing drug labels from paper and packaging. Those were bought by the pharmacy. If it is not charged to the buyer, from the management point of view, how can we cover these cost (Pharmacist, FGD 3)

Such extra burden diminished the motivation for pharmacies to keep participating in the partnership. Some pharmacies had to terminate the partnership because the costs of participation outweighed the benefit.

I have once taken drugs from DHO, but the reporting and requirements for requesting drugs are complicated, so I do not participate in the initiative anymore. I heard many complaints from other pharmacists that the process to request drugs is complicated and time-consuming. I don’t want that it will become problem in the future, so I stop taking drugs from DHO (Pharmacist 7, interview)

#### Opportunity costs

Although the majority of pharmacist participants had a positive attitude towards the partnership, some participants expressed concerns that they received almost no tangible benefit from participation. Instead, participating in this partnership often created significant opportunity costs for pharmacies due to the extra time invested for reporting and procurement process.

The frequent stock-out of antimalarial drugs in the district warehouse even led some pharmacies to no longer sourced antimalarials from the DHO because that time invested for procuring the medicine using the partnership mechanism was perceived as too much.

The previous pharmacist said that the procedure is complicated because several she times requested drugs to DHO, they said they were out of stock. So, because we need malaria drugs, we procured other malaria drugs ourselves (Pharmacist, FGD 1)

However, other pharmacy participants mentioned that participating in this partnership could result in increased revenue for pharmacies, because patients would also buy other drugs such as analgesics or antipyretics.

Physicians mentioned that although providing ACTs alone did not offer any profit for the pharmacies, they could gain income from physician consultation and laboratory examination fees.

...we got profit from other income, such as consultation fee, administration fee, other drugs selling, laboratory test. How much can we get from selling ACTs? We don’t want to burden the patient. Furthermore, it aims to eliminate malaria. (Physician 2, interview)

Community pharmacies that had a partnership with the National Health Insurance (BPJS) gained some financial benefit by joining in the partnership because they did not need to procure antimalarials using capitation payment. Hence, they would save some money from the drug procurement budget.

I said [to the doctor] that we could no longer procure drugs by ourselves because of the high price. Most of our patients were covered by the National Health Insurance (BPJS). Therefore, we would better take drugs from DHO. It will be really helpful because we could save the budget for buying drugs. It is free; it will benefit us (Pharmacist, FGD 2)

Non-monetary benefits such as opportunities for continuing education were also mentioned. For example, laboratory analysts were given training on parasitological examination, while physicians and pharmacists were given malaria case management seminars.

#### Ethicality

Participants raised a number of ethical issues in the initiation and implementation of the partnership. First, the nature of participation in the partnership  was supposed to be voluntary. However, the initiation of the partnership was not clearly communicated to the community pharmacies. One participant from community pharmacies mentioned that at first, they were invited to a meeting but were not informed that the meeting was aimed at signing the MoU of the partnership. As a result, the participant felt that they had little choice other than participating in the partnership.

... when we got there, it was a meeting on a partnership, and we didn’t know that. So, we were already in the meeting; it’s impossible if we reject to participate… (laughing). The only thing we can do was accepting and trying to get involved. (Pharmacist 2, interview)

Moreover, the government's dual role might create an undue influence on community pharmacies to participate in the partnership. On the one hand, the DHO was the initiator of the partnership and had a great interest in achieving the partnership's goal. On the other hand, the DHO was also authorized to recommend the issuance or renewal of an operational license for community pharmacies. This may impact the voluntary nature of participation in the partnership because community pharmacies might fear that non-participation would affect their licensing processes.

The second issue is the restriction of the sale of patent drugs by pharmacies. One requirement of the partnership was that participating pharmacies could not dispense patent antimalarial drugs and non-ACT drugs. By law, however, despite being the highest health authority in the district, the DHO did not have the rights to restrict the sale of certain drugs by the pharmacies, as long as these drugs have been issued permits by the Food and Drug Administration to be distributed in Indonesia. Therefore, the provision of non-ACT and patented drugs at pharmacies cannot be prohibited.

In addition, DHO also realized that strictly imposing the rules of restricting the sale of certain drugs at pharmacies would potentially reduce the already small revenue of pharmacies. This has created moral conflicts among DHO participants.

....we actually tried that chloroquine not being sold anymore (at pharmacies), but we cannot prohibit them because of economic factor (DHO, interview)

The third is the issue of charging administrative fees to patients. Pharmacist participants who also sat in the professional association mentioned that it is not appropriate to charge patients even though it is only a small amount of money because antimalarials are provided for free by the central government. Selling the drugs at some costs, even though it is for administrative costs, could pose legal problems to the health authorities.

Even though it is only a small amount of money, we have to look at the MoU. Why we agreed with IDR 5000. If we look at the MoU, we are wrong. We cannot charge the patient. This drug (ACT) is from the government. It is free. Someday if there is someone who looks at the details on this matter, we could be blamed and it can be a problem..... We can be sued in Administrative Court because this is wrong. OK, it might be a consensus, but we cannot charge for money. (Pharmacist, FGD 2)

Concerns were also raised that the provisions of ACTs drugs at no cost in pharmacies might lead to practices violating the MoU among the pharmacy staff. This led to some pharmacies refused to participate in the partnership.

We do not take drugs from DHO because we are worried that the drugs might be sold by our staffs. It will affect our reputation. (Pharmacist 4, interview)

Because of these potential conflicts, it was decided in a consensus that patients should not be informed that antimalarial drugs are provided at no cost in pharmacies. Pharmacist participants also mentioned that they sometimes had to lie to patients who requested antimalarial drugs without prescriptions. Instead saying that the medicine has to be purchased with a prescription, they said that the medicine is out of stock. Because of the sociocultural characteristics of the local people, rejection to a patient’s request would potentially create anger among clients.

…Furthermore, we live in Papua, we would rather say that we do not have the drugs than saying to them that they have to bring a prescription to buy it. It will trigger anger toward us (Pharmacist 3, interview)

In line with that, all client participants did not know that antimalarials are provided for free at pharmacies.

I have heard from my neighbor about this free malaria drugs. I heard that we could get free malaria drug from hospital or PHC, but I never heard that it is also available at community pharmacies (Patient 3, interview)

Because not all pharmacies participate in the partnership, the practice of dispensing antimalarials at no cost was not universally implemented in all pharmacies. As a consequence, patients who accessed antimalarials at non-participating pharmacies might have to pay higher costs than those who accessed at the participating pharmacists, raising inequity in access issues.

However, there are pharmacies selling antimalarials for some price. It led confusion among patients, why it was sold when we could get it for free from PHC. (Pharmacist, FGD 3)

## Discussion

This is the first study investigating the acceptability of public–private partnership in malaria control as perceived by different stakeholders in Manokwari, a malaria-endemic, semi-urban district in West Papua, Indonesia. The partnership focuses on the provision of subsidized ACTs in community pharmacies based on diagnostic confirmation. Although public–private partnership has been one of the national malaria control strategies, the approach of providing subsidized ACTs in pharmacies is quite new in Indonesia. Therefore, our study findings are an important contribution to developing a strategy to increase access to ACTs in Indonesia and other countries with similar settings.

Our study found that the provision of ACTs at no cost in community pharmacies was perceived as effective and created positive attitudes among the key partners involved, which perhaps stemmed from the perceived effectiveness of the intervention. The increase in the use of ACTs reported by pharmacies suggested that the partnership has the potentials to expand access to ACTs through community pharmacies and physicians. Prior to the partnership, as also found in other endemic countries [3], much of the provision of antimalarials in pharmacies and other drug sellers were unregulated. In addition to that, only patent non-ACT antimalarials, such as quinine are available. Previous studies showed that distributing subsidized ACTs through pharmacies and drug outlets results in a substantial increase of ACT use in the private sector because of improved availability and affordability [2, 23, 24].

Furthermore, as ACTs are only given for laboratory-confirmed malaria, the partnership also helps to ensure access to quality malaria diagnosis and treatment, especially with fairly good intervention coherence in this study. Experience with similar initiatives in other countries also showed that community pharmacies and physicians are able to provide a standard of care that previously only existed in the public sector, thus improving access to prompt and effective treatment in the community [25, 26]. In Lao PDR, the provision of malaria diagnostic and treatment services in registered local pharmacies and clinics contributed to a significant increase in the proportion of patients tested for malaria [27]. In Ethiopia, a mix between the public and private provision of malaria care services has significantly improved adherence to treatment guidelines [28]. However, physicians' involvement in the present partnership should be strengthened to ensure adherence to treatment guidelines.

Benefits offered for the private providers in this program were similar to those applied in other countries, including continuing education for private health providers [27] and profits from OTC sales. Nevertheless, the tangible benefits of the partnership were deemed too small compared to the burden. The perceived burdens associated with the partnership's participation, such as time, human resources, and opportunity costs, were quite substantial. The perceived burden and inherent interest in revenue and profit can conflict with the positive public health impacts of the partnership, and if the negative outweighs the positive, the partnership can be terminated by the private health providers. Experience from other countries showed that offering benefits to the private sector could increase retention in the program. In Tanzania, formally recognizing businesses as an “Accredited Drug Dispensing Outlet” was the main reason for owners to participate in public–private partnerships [23].

A number of ethical issues raised by the participants should be a matter of concern for the program implementers. Brinkerhoff [29] asserted that in government and private partnerships, jointly determined goals, collaborative and consensus-based decision-making, non-hierarchical and horizontal structures and processes, and synergistic interactions among partners should be in place in order to build a long-term relationship. Despite the common goal of malaria elimination, a low degree of a mutual relationship was still observed in the present partnership. Private health providers, as the key partner in the partnership should be adequately informed about the procedure and consequences pertinent to participation in the partnership, as well as their individual and collective performance on a regular basis. Such involvement will augment the motivation of private health providers to commit to the partnership and contribute their resources to achieve the common goal.

Moreover, inequity in access to ACTs may exist, partly because of the programmatic issues. The low coverage of the partnership despite the growing number of pharmacies leads to different antimalarial dispensing behavior between participating and non-participating pharmacies. The lack of involvement of other private health providers, especially physicians, in the partnership, resulted in different practices when prescribing antimalarials. The absence of written guidelines also caused differences in ACTs dispensing practices between participating pharmacies, especially when the medication is out of stock. Consequently, patent ACTs and non-ACTs such as quinine were still provided at cost and sometimes dispensed without a positive diagnosis of malaria, creating inequity in access to standardized treatment among clients of community pharmacies.

### Strength and limitations

Our study provides insights into the perceptions of different stakeholders experiencing and practicing the partnership. The study, however, did not involve newly established pharmacies in the study location. Thus, we were not able to document their perspectives towards this program. Most of these providers were established between 2018 and 2019, during which the MoU was already established and ongoing. The recruitment of study participants was also halted because of the emergence of the COVID-19 pandemic. Therefore, the number of patients interviewed was only a few, especially those from the non-participating pharmacies. The study  reflected Manokwari's situation, which has different sociocultural and economic characteristics from other areas in Indonesia. The behavior of private health providers in this area may differ from those in other areas.

### Implications

For the partnership to be sustained, the overall procedure and implementation of the partnership should first be revisited. First, the growth in the number of community pharmacies should be considered in expanding the partnership to ensure equity in access to ACTs among clients of pharmacies could be narrowed down. First, the growth in the number of community pharmacies should be considered in expanding the partnership to ensure equity in access to ACTs among pharmacies' clients. Information on the partnership needs to be disseminated to new pharmacies to ensure high coverage. Second, programmatic issues such as the shortage or stock-out of drugs and the absence of staff responsible for procurement should be addressed to ensure the quality of the program.

Third, it is important to aim for a balance between public health benefits and business issues in the implementation of the partnership for ACTs distribution. Adequate incentives for participating providers should be provided. If monetary incentives cannot be offered, the partnership should consider including non-monetary yet tangible benefits for private providers, for example, a regular capacity building workshop in malaria diagnosis and treatment.

Lastly, to improve the quality of the program including adherence to the malaria diagnosis and treatment guideline, an implementation procedure should be developed and disseminated. Supervision and monitoring to private health providers should be conducted on a regular basis to identify bottlenecks in the implementation and find a solution.

## Conclusion

The acceptability of public–private partnerships for the provision of ACTs among private providers in Manokwari was still low. Despite the perceived effectiveness, the burden of participation in the long run might outweigh the benefits, posing threats to the intervention's sustainability. Innovations to simplify the partnership procedures in combination with performance-based incentives are needed to improve the implementation. Engagement of patients and physicians is needed to increase the partnership's coverage and effectiveness, thus ensuring improved access to effective diagnosis and treatment of malaria.

## Data Availability

Data and materials supporting the findings are available from corresponding author on reasonable request.
